# Discovery of Resistance Genes in Rye by Targeted Long-Read Sequencing and Association Genetics

**DOI:** 10.3390/cells11081273

**Published:** 2022-04-09

**Authors:** Nikolaj M. Vendelbo, Khalid Mahmood, Burkhard Steuernagel, Brande B. H. Wulff, Pernille Sarup, Mogens S. Hovmøller, Annemarie Fejer Justesen, Peter S. Kristensen, Jihad Orabi, Ahmed Jahoor

**Affiliations:** 1 Department of Molecular Breeding, Nordic Seed A/S, 8300 Odder, Denmark; khma@nordicseed.com (K.M.); pesa@nordicseed.com (P.S.); pskr@nordicseed.com (P.S.K.); jior@nordicseed.com (J.O.); ahja@nordicseed.com (A.J.); 2 Department of Agroecology, Faculty of Technology, Aarhus University, 4200 Slagelse, Denmark; mogens.hovmoller@agro.au.dk (M.S.H.); annemariefejer.justesen@agro.au.dk (A.F.J.); 3 John Innes Centre, Norwich Research Park, Norwich NR4 7UH, UK; burkhard.steuernagel@jic.ac.uk (B.S.); brande.wulff@kaust.edu.sa (B.B.H.W.); 4 Plant Science Program, Biological and Environmental Science and Engineering Division, King Abdullah University of Science and Technology (KAUST), Thuwal 23955-6900, Saudi Arabia; 5 Center for Desert Agriculture, King Abdullah University of Science and Technology (KAUST), Thuwal 23955-6900, Saudi Arabia; 6 Department of Plant Breeding, The Swedish University of Agricultural Sciences, SE-234 22 Alnarp, Sweden

**Keywords:** *Secale cereale* L., leaf rust, brown rust, *Puccinia recondita* f. sp. *secalis*, nucleotide-binding leucine-rich repeat (NLR), resistance gene enrichment sequencing (RenSeq), single-molecule high-fidelity sequencing (HiFi), genome-wide association study (GWAS), *k*-mer

## Abstract

The majority of released rye cultivars are susceptible to leaf rust because of a low level of resistance in the predominant hybrid rye-breeding gene pools Petkus and Carsten. To discover new sources of leaf rust resistance, we phenotyped a diverse panel of inbred lines from the less prevalent Gülzow germplasm using six distinct isolates of *Puccinia recondita* f. sp. *secalis* and found that 55 out of 92 lines were resistant to all isolates. By performing a genome-wide association study using 261,406 informative SNP markers, we identified five resistance-associated QTLs on chromosome arms 1RS, 1RL, 2RL, 5RL and 7RS. To identify candidate *Puccinia recondita* (*Pr*) resistance genes in these QTLs, we sequenced the rye nucleotide-binding leucine-rich repeat (NLR) intracellular immune receptor complement using a Triticeae NLR bait-library and PacBio^®^ long-read single-molecule high-fidelity (HiFi) sequencing. Trait-genotype correlations across 10 resistant and 10 susceptible lines identified four candidate NLR-encoding *Pr* genes. One of these physically co-localized with molecular markers delimiting *Pr3* on chromosome arm 1RS and the top-most resistance-associated QTL in the panel.

## 1. Introduction

Leaf rust is one of the most devastating diseases in hybrid rye (*Secale cereale* L.) as it can reduce grain yield by up to 27% in the field [1]. Because the predominant heterotic gene pools used for the breeding of rye hybrids, ‘Petkus’ and ‘Carsten’, suffer from low inherent resistance, most newly released cultivars are highly susceptible to leaf rust (Supplementary Table S1) [2,3]. In rye, leaf rust is caused by the heteroecious macrocyclic fungal basidiomycete, *Puccinia recondita* f. sp. *secalis* (*Prs*), which is prolific across the rye growing regions in Europe [4]. Unlike wheat leaf rust, stripe rust and stem rust, the alternate plant host of *Prs*, small bugloss (*Anchusa arvensis* L.), is widespread in Northern European flora and a common weed in agricultural fields [5,6,7,8]. The evolutionary plasticity generated by potential frequent sexual recombination and the capacity of spores to migrate long distances accentuate the risk of novel, aggressive *Prs* pathotypes emerging and spreading, which in turn constitutes a serious threat to hybrid rye production [4,9,10,11].

Host resistance represents a sustainable and more environmentally conscious alternative to chemical control strategies [12]. Currently, six major leaf rust resistance (*R*) genes have been identified in rye: *Pr3* (on the short arm of chromosome 1, 1RS), *Prs4* (on the long arm of chromosome 1, 1RL), *Pr5* (1RL), *Pr1* (6RL), *PrNOS1* (7RS) and *Pr2* (7RL) [13,14,15]. In addition, three major leaf rust *R* genes have been described in translocation lines between rye and wheat (*Triticum aestivum* L.), with rye as the resistance donor and designated according to the wheat gene nomenclature as *Leaf rust resistance 26* (*Lr26*, from translocation 1BL-1RS), *Lr25* (4BS.4BL—2RL) and *Lr45* (2AS—2RS.2RL) [16,17]. The majority of *R* genes encode intracellular nucleotide-binding leucine-rich repeat (NLR) immune receptor proteins [18]. In the grasses, canonical NLR genes are comprised of three domains, (i) a C-terminal leucine-rich repeat (LRR) domain for pathogen effector recognition and NLR autoinhibition [19,20]; (ii) a conserved nucleotide-binding (NB) domain in the middle of the protein that is involved in regulation of NLR activation [21]; and (iii), an N-terminal coiled-coil (CC) domain mediating the downstream immune-signaling cascade [22]. In the reference genome of the German inbred grain-type rye line Lo7, 1167 NLR genes have been reported, with this number reaching 1447 in the Chinese forage-type Weining [23,24].

The breeding of novel, resistant cultivars has been accelerated by the adoption of genomic and molecular breeding techniques. The use of marker-assisted selection (MAS) allows the pyramiding of *R* genes and intermediate-effect quantitative trait loci (QTLs) for enhancing the durability of resistance [25,26,27]. When coupled with speed-breeding approaches, MAS enables the rapid development of novel cultivars by the introgression of exotic *R* genes into an elite background [28,29]. Importantly, mining for novel genetic variation for resistance against a given pathogen is a prerequisite for the continuous development of novel resistant cultivars. Next-generation mapping resources such as nested association mapping (NAM) and multiparent advanced generation intercross (MAGIC) populations, in combination with high-density single-nucleotide polymorphism (SNP) genotyping, have provided powerful techniques for the identification of *R* genes and QTLs [30,31]. Historical recombination events naturally present within a diverse panel for a crop species can also be exploited for mining *R* genes and QTLs through genome-wide association studies (GWAS) and SNP genotype data [32]. GWAS identify a genomic region controlling a trait of interest and, predominantly, a nonfunctional marker associated with the trait that can be readily implemented in MAS [33]. However, as the markers often reside outside the actual genes of interests, how informative they are can be compromised by crossover events. Furthermore, the identification of the causal gene is often not possible in cases when the *R* gene is absent from the reference genome or exhibits considerable divergence from the reference sequence [34].

The limitations of GWAS can be overcome by performing trait association on sub-sequences (*k*-mers) derived from *R* gene enrichment sequencing (AgRenSeq). To obtain trait-genotype associations in cis, the associated *k*-mers are mapped to the contigs of a de novo assembly of a resistant accession [35]. AgRenSeq makes it possible to mine for novel *R* genes in undomesticated wild relatives of crop species without the need for a high-quality reference genome. Instead of identifying a resistance-associated genomic region, RenSeq identifies candidate NLR genes conferring resistance, from which functional markers can be developed for MAS and as a resource for genetic engineering [36]. However, the de novo assembly of NLR genes from short read data is a complex task due to their high copy number and sequence similarity between paralogs [37]. This limitation was previously alleviated by coupling RenSeq with long-read PacBio^®^ single-molecule real-time (SMRT) sequencing [38,39]. Since then, improvements to the PacBio^®^ HiFi sequel II system have demonstrated 99.9% accuracy in the recall rate of single-nucleotide variants (SNVs) and 96% accuracy for insertions and deletions (InDels) [40].

Currently, RenSeq has been deployed in Arabidopsis (*Arabidopsis thaliana*), strawberry (*Fragaria* × *ananassa*), potato (*Solanum tuberosum* L.), tomato (*Solanum lycopersicum* L.) hexaploid bread wheat, an introgression line between wheat and mosquito grass (*Dasypyrum villosum* L.) and the wheat wild relatives Tausch’s goatgrass (*Aegilops tauschii*) and goat grass (*Ae. peregrina*) [35,38,39,41,42,43,44].

The objective of this study was to determine the efficacy of SMRT AgRenSeq for the mining of leaf rust *R* genes in rye using a panel of restorer lines recently demonstrated to exhibit a high level of resistance in the field [15].

## 2. Materials and Methods

### 2.1. Collection of Danish Puccinia Recondita f. sp. Secalis Samples, Their Multiplication and Single Pustule Isolation

With no molecular markers or differential sets available for the identification of races in *Puccinia recondita* f. sp. *secalis* (*Prs*), a national sampling of the pathogen was conducted at six geographically distinct sites in Denmark from 2018 to 2019 (Supplementary Table S2, Supplementary Figure S1). Multiplication of field samples and isolation of single-pustule isolates (SPI) were performed as described by Vendelbo et al. [15].

### 2.2. Plant Materials and DNA Extraction

In total, 92 inbred rye (*Secale cereale* L.) restorer lines belonging to the elite hybrid rye-breeding germplasm at Nordic Seed A/S (Dyngby, Denmark) were selected for the study. Four seeds per line were sown per well in 104-well trays containing a fine-grained sphagnum substrate. The seedlings were propagated in the greenhouse facilities at Nordic Seed A/S under 16 h of daylight at 18–24 °C and 8 h of darkness at 14–16 °C. The lowest sections of two coleoptiles and primary leaves were excised after 7 days (equivalent to 75 mg of plant material) and placed in a 96-well Micro-Dilution Tube System (STARLAB International GmbH) containing two 4 mm glass beads per 1.2 mL tube. Plant tissue samples were stored at −20 °C for 2 days prior to freeze-drying for an additional 2 d. DNA extraction was performed using an adapted SDS-based method according to USDA [45] according to Pallotta et al. [46]. The DNA concentration and 260/280 nm absorption ratio were measured using an Epoch ^TM^ microplate spectrophotometer (Biotek^®^ Instruments, Winooski, V.T., USA). Fragmentation of genomic DNA was assessed by size separation on a 1.2% (*w*/*v*) agarose gel.

### 2.3. Phenotyping for Pathogen Resistance

All lines were phenotyped for their resistance response against six geographically distinct *Prs* SPIs to investigate the level of resistance to leaf rust in the panel. Phenotyping was conducted using a 10-step (0–9) infection-type (IT) scale adapted by McNeal et al. [47] and Hovmoller et al. [48]. Seedlings with a mean IT of 0–2 were categorized as ‘resistant’, IT of 3–4 as ‘partially resistant’, IT of 5–6 as ‘partially susceptible’ and IT of 7–9 as ‘susceptible’. Large-scale multiplication of trial inoculum was done in spore-proof greenhouse cabins at Nordic Seed A/S (Dyngby, Denmark) in accordance with the protocol described by Vendelbo et al. [15]. In the trial, eight seeds per line were sown in a 28-hole tray containing a coarse-grained sphagnum substrate. The trial followed a partially randomized design with two repetitions for each of the two trial replicates, hence producing four scorings per line per SPI evaluated.

At 14 days after sowing, each tray was inoculated with 30 mg of SPI spores solubilized in 4 mL 3M ™ Novec ™ 7100 (Sigma-Aldrich^®^, St. Louis, MO, USA) engineering fluid according to the method described by Thach et al. [49]. After incubation for 14 d, the lines were phenotyped for their infection-type response by scoring both the first and second leaf of each of the eight seedlings per pot.

### 2.4. Molecular Marker Resource and SNP Genotyping

For each line, 200 ng of high molecular-weight genomic DNA with a 260/280 nm absorbance ratio of ≥1.8 were sent for SNP genotyping at Eurofins Genomics Europe Genotyping (Aarhus, Denmark). Genotyping was accomplished using a 600 K SNP array with 600,843 SNP markers on an Affymetrix GeneTitan ™ Scanner platform (Thermo Fisher Scientific, Waltham, MA, USA) [50]. Mapping of the SNP markers of the Lo7 rye reference genome and characterization of its performance in the assayed germplasm was recently investigated by [51]. The marker map is available at https://doi.org/10.5281/ZENODO.5086235 (accessed on 2 January 2022).

### 2.5. Data Analysis

Bioinformatics analysis was performed with the R Studio (v. 1.3.959) interface with R statistical software (v. 4.0.1) with various predesigned packages [52,53]. Prior to analysis of the SNP genotyping data, markers were filtered for marker allele frequencies of at least 5%, missing individual scores below 20% and missing marker scores below 10% to identify informative markers. Computationally demanding tasks were run at the national, high-performance computing facility GenomeDK at the Aarhus Genome Data Center, Aarhus University. Custom R scripts for all visual outputs have been provided as supporting information.

### 2.6. Genome-Wide Association Study

The exploration of SNPs associated with leaf rust resistance relied on a genome-wide association study (GWAS) using the genomic-association and prediction-integration-tool (GAPIT v. 3) package in R [54]. The phenotypic input for GWAS included all individual recordings for the six SPI *Prs* scorings, averaged across the two replicates.

### 2.7. In Silico Test of the Bait Library

Before using the 600 K Triticeae nucleotide-binding leucine-rich repeat (NLR) bait-library (Tv_1) designed by Steuernagel et al. [42], an in silico test was run to evaluate its capture success with rye. Baits were mapped to the rye reference-genome Lo7 NLR repertoire using the Basic Local Alignment Search Tool for nucleotide (BLASTN v. 2.9.0+) function at the National Center for Biotechnological Information (NCBI) [55]. Bait hits were then filtered for a minimum of 80% sequence identity over the full 120-nt bait length, according to the findings made by Jupe et al. [37], who reported that ~80% sequence identification between bait and NLR sequence is sufficient for enrichment. The Tv_1 bait library is available at https://github.com/steuernb/MutantHunter/ (accessed on 7 August 2021). The bait library was assessed using custom scripts and inherent functions in R for NLR sequence capture success, unique baits per NLR sequence, unique NLR sequences per bait, distribution of bait alignment (in bp) and alignment identity (in %).

### 2.8. Phylogenetic Analysis and Pairwise Selection of Restorer Lines

To reduce the false association from noncausative background, 10 pairs of lines were selected for Single-Molecule Real Time *R* gene enrichment and Sequencing (SMRT RenSeq), each comprising two phylogenetically closely related lines but exhibiting a divergent resistance profile. Phylogenetic analysis was performed with a neighbor-joining clustering of lines using measures of their Euclidean genetic distance using the ape (v. 5.3) R package [56]. The tree was constructed after 10,000 bootstrapping iterations with weak nodes showing less than 80% recurrence collapsed into multifurcations. The tree was drawn with iTOL (v. 6.1.1) (http://itol.embl.de/ (accessed on 7 August 2021)), allowing a color-based visualization of the resistance response presented by each line against the six *Prs* SPIs as concentric circles [57].

### 2.9. Single-Molecule Real-Time High-Fidelity Resistance Gene Enrichment Sequencing

The construction of PacBio^®^ RenSeq libraries was outsourced to Arbor Biosciences (M.I., USA). A minimum of 5 µg per sample of genomic DNA demonstrating a UV 260/280 absorbance ratio between 1.7 and 1.9 and of at least 10 kb modal length was provided. Genomic DNA libraries were enriched for NLR sequences using the Tv_1 60 K Triticeae NLR bait-library [42]. The enriched target DNA was sequenced on a PacBio^®^ Sequell II long-read SMRT platform according to PacBio^®^ methods without modifications to generate 1.5 Gb of high-fidelity (HiFi) circular consensus sequence (CCS) per line. Prior to sequencing, DNA libraries were multiplexed to allow pooling.

### 2.10. De Novo Assembly and NLR Annotation

The CCSs were assembled with HiCanu (v. 2.0; -pacbio-hifi, trimReadsCoverage = 2, errorRate = 0.01, genomeSize = 8.8 m, minOverlapLength = 500, minReadLength = 1000) [58]. The expected genome size of the rye NLR repertoire was estimated using NLR data from the recent rye reference genome Lo7 [23] with the addition of a 40% flanking region in accordance with the study by Van de Weyer et al. [41]. Contigs associated with NLR genes were annotated using NLR-Parser (v. 3) and NLR-Annotator (https://github.com/steuernb/NLR-Annotator (accessed on 7 August 2021)) [59,60]. NLR contigs were mapped to the rye reference genome Lo7 using NCBI BLASTN (v. 2.9.0+) with a significance threshold set to 1 × 10^−5^, selecting the physical position of the top hit [55]. Contig positions were kept if they met the criteria of ≥90% alignment length and ≥85% alignment identity.

### 2.11. In Vitro Test of the Bait Library

To investigate Tv_1 bait-library performance in vitro, the individual restorer line SMRT RenSeq data were subjected to a similar analysis to that described for the in silico test. In addition, to assess the bait distribution amongst lines, baits were classified into three categories: (i) ‘core’, aligning in more than 12 lines; (ii) ‘common’, aligning in 5–12 lines; and (iii) ‘rare’, aligning in fewer than 5 lines. The distribution of bait alignment, bait alignment identity and bait category distribution were visualized using the ggplot2 (v. 3.3.3) R package [61]. The distributions of baits per NLR contig and NLR contigs per bait were visualized using the YaRrr R (v. 0.1.5) package. The R scripts for the in silico and in vitro assessment of the 60 K bait library on the panel are provided as supporting information.

### 2.12. K-Mer Presence/Absence Matrix

Raw CCS reads were processed into *k*-mers (*k* = 51 nt) using Jellyfish (v. 2.2.10), discarding rare *k*-mers with a count ≤10 per line [62]. A collective *k*-mer presence/absence matrix was generated by pooling *k*-mers from all lines in a binary format with 1 (‘present’) and 0 (‘absent’). The matrix was filtered for rare *k*-mers present in ≤3 lines. *k*-mer processing of the CCS reads was performed following the Java source code published at https://github.com/steuernb/AgRenSeq (accessed on 7 August 2021).

### 2.13. Association Genetics RenSeq Analysis

The phenotypic data were converted using the following formula developed for phenotypic scores in Stackman’s IT scale (−2 to 2).

(1) $${\mathrm{Stackman}}^{’}s\;\mathrm{IT}\;\mathrm{score}=2-\left(\frac{4}{9}*Severity\;Score_{\;0-9}\right)$$

Identification of candidate leaf rust resistance genes amongst the NLR-annotated contigs was performed by AgRenSeq analysis following the Java pipeline provided by Arora et al. [35] at https://github.com/steuernb/AgRenSeq (accessed on 7 August 2021) and the general linear regression model (AgRenSeq-GLM) using the Python module provided at https://github.com/kgaurav1208/AgRenSeq_GLM (accessed on 19 August 2021). This modulated analysis utilizes a linear regression model for each of the *k*-mers to account for population structure amongst lines and to likelihood-test for nested models to output a *p*-value for each of the *k*-mers’ association with resistance. A standard Bonferroni-corrected threshold of α = 0.025 was used as the significance threshold.

### 2.14. Characterization of the Candidate Leaf Rust Resistance Gene Pr3

To improve NLR annotation, a manual reference assembly was generated for all leaf-rust-resistance-associated contigs identified by AgRenSeq to expand the contig size. For each contig, raw CCS mapping to the contig was performed using NCBI BLASTN (v. 2.9.0+) function, selecting CCS aligning over at least 2000 bp and with 95% sequence identity [55]. CCS reads were trimmed for adaptor sequences and assembled using the respective leaf-rust-resistance-associated contig as reference and manually inspected to generate a consensus sequence in Geneious Prime (v. 2020.2.3) [63]. The gene structure of the manually assembled consensus NLR contigs was predicted using the AUGUSTUS (3.4.0) program [64]. The prediction of protein domain structures was performed using InterPro-Scan [65]. Prediction of LRR motifs was performed using LRRpredictor (v. 1.0) [66]. Coding sequences were mapped to the Lo7 and Weining reference genomes using NCBI BLASTN (v. 2.9.0+) [23,24,55].

To distinguish unique NLR genes from homologs within the panel of leaf-rust-resistance-associated contigs identified by AgRenSeq, a phylogenetic analysis was undertaken on the basis of protein sequence similarity using the pipeline developed by Toparslan et al. [67] in R. Multiple sequence alignment of protein sequences was performed using ‘Clustal Omega’ in the msa (v. 1.20) R package. A neighbor-joining tree was constructed using Nei’s standard genetic distance and visualized using the ggtree (v. 2.2.4) R package [68]. To validate the tree, 10,000 bootstrapping iterations were run. As reference, sequences of known NLR genes conferring rust resistance in cereals were included in the analysis, obtained from the UniProt and NCBI online databases [55,69].

Identification of single-nucleotide variants (SNVs) and insertions/deletions (InDels) between candidate gene variants was performed by multiple sequence alignment using the Multiple Sequence Comparison by Log-Expectation (MUSCLE) method in Geneious Prime (v. 2020.2.3) with ≤10 iterations. For comparative analysis, NLR genes at mapping positions in the Lo7 and Weining reference genomes were extracted from the NLR annotation file provided by [51]. Annotation files are available at https://doi.org/10.5281/zenodo.5085854 (accessed on 10 November 2021). If present in the reference genomes (≥95% sequence similarity), coding sequences and de novo, predicted protein sequences were extracted. To investigate whether the candidate *Pr* gene was positioned near designated *Pr* genes on chromosome arm 1RS, flanking co-segregating markers were extracted and mapped to the Lo7 and Weining reference genomes by BLASTN [14,70].

### 2.15. Graphical Editing

Graphs and Figures were saved from R in .svg format and manually curated using the Inkscape (v. 1.1) program (https://inkscape.org/ (accessed on 7 August 2021)).

## 3. Results

### 3.1. Phenotyping of Rye Breeding Lines for Resistance to Leaf Rust

**Figure 1:**
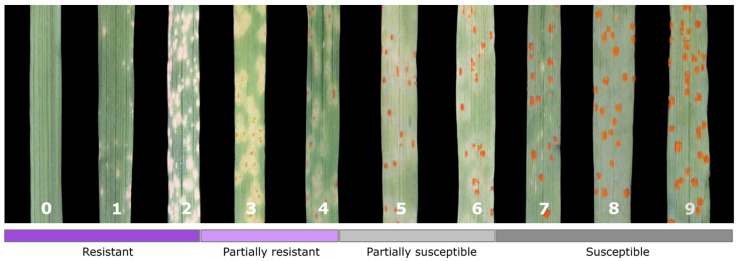
Infection-type (IT) response scale for leaf rust in rye (*Secale cereale* L.) caused by the fungal pathogen *Puccinia recondita* f. sp. *secalis* according to Hovmøller et al. (2017) and McNeal et al. (1971). In terms of virulence/avirulence, IT 0–6 are considered ‘avirulent’ and 7–9 as ‘virulent’.

We tested 92 inbred rye restorer breeding lines for leaf rust resistance using six *Puccinia recondita* f. sp. *secalis* (*Prs*) single-pustule isolates (SPIs) of distinct geographical origin, which uncovered a high level of resistance in the germplasm (Supplementary Table S2, Supplementary Figure S1). We categorized 51 lines as consistently resistant (IT 0–2), 11 as partially resistant (IT 3–4), 11 as partially susceptible (IT 5–6) and the remaining 19 as consistently susceptible (IT 7–9) (Supplementary Table S3). Four lines showed an SPI-specific resistance response. Figure 1 illustrates the 10 infection types as a resource for future studies of leaf rust resistance in rye.

### 3.2. Genome-Wide Association Study

**Figure 2:**
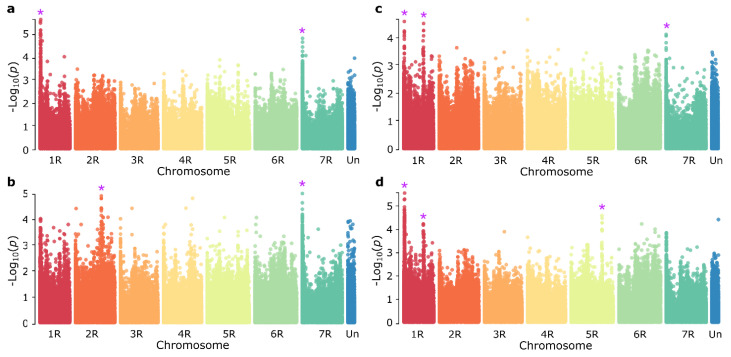
Manhattan plots for genome-wide association study (GWAS) for leaf rust resistance in 92 inbred rye lines. GWAS was performed using 261,406 informative SNP markers mapped to the Lo7 reference genome. The entire germplasm collection was phenotyped for resistance against six distinct *Puccinia recondita* f. sp. *secalis* single-pustule isolates (SPIs) in a greenhouse in two replicate trials. (**a**) SPI-5, (**b**) SPI-3, (**c**) SPI-1 and (**d**) SPI-2. Five genomic regions associated with resistance are marked by magenta asterisks. The Bonferroni-adjusted significance threshold based on informative markers was 6.72.

Quality filtering of markers from the 600 K SNP array allowed the identification of 261,406 informative markers (Data File S1). To explore the genetic basis underlying leaf rust resistance in the panel, we performed a genome-wide association study (GWAS) across the six *Prs* SPIs (Supplementary Figure S2, Supplementary Table S4). We identified five genomic positions associated with leaf rust resistance against one or more SPI that mapped to chromosome arms 1RS, 1RL, 2RL, 5RL and 7RS (Table 1, Figure 2). However, none of the markers exhibited a *p*-value above the Bonferroni significance threshold of −Log_10_(*p*) = 6.72. Each marker explained between 9.3 and 13.1% of the phenotypic variance (Table 1).

### 3.3. In Silico Test of 600 K Triticeae NLR Bait-Library for Rye

Prior to RenSeq analysis, we assessed the performance of the 600 K Triticeae-specific NLR bait-library Tv_1 in rye by performing an in silico analysis of its capture success rate against the NLR repertoire of the Lo7 reference genome. Using a set threshold, we determined that 29,785 baits align, capturing 1125 out of the 1167 annotated NLR sequences in the Lo7 reference (Supplementary Table S7). Each bait mapped, on average, to 5 ± 6 (standard deviation, SD) unique NLR sequences, with a mean of 140 ± 164 (SD) unique baits mapped per NLR sequence (Supplementary Table S5).

### 3.4. Phylogenetic Analysis and Pairwise Selection of Restorer Lines

**Figure 3:**
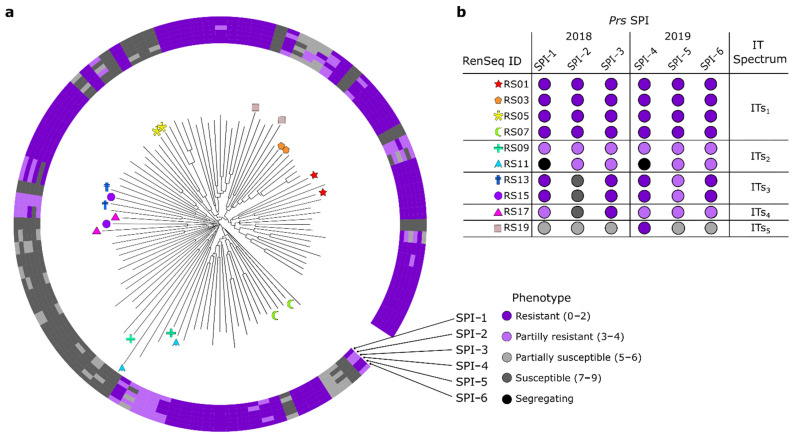
Phylogenetic analysis and type of leaf rust infection response in 92 inbred rye lines. (**a**) Circular neighbor-joining dendrogram indicating the infection types (IT) against six geographically distinct Danish *Puccinia recondita* f. sp. *secalis* single-pustule isolates, shown as concentric circles. Ten pairs, comprising two closely related lines with divergent ITs, selected for SMRT RenSeq are marked by stars. (**b**) IT spectrum of the 10 resistant lines. The 10 susceptible lines are not depicted. They all had IT scores of 7 to 9 (Supplementary Table S3).

To identify *Pr* genes in the panel, we reasoned that selecting pairs of lines that are closely related but differ only in their resistance profile would maximize the discriminatory power of informative polymorphisms without having to resort to sequencing the entire panel (Figure 3a). The corresponding shortlisted resistant lines exhibited five different infection-type (ITs) spectra: resistant (ITs_1_), partially resistant (ITs_2_) and three different SPI-specific spectra (ITs_3–5_) (Figure 3b).

### 3.5. SMRT RenSeq, De Novo Assembly and NLR Annotation

To increase the contiguity and accuracy of the assembled NLRs, we sequenced the selected lines using PacBio^®^ HiFi technology. SMRT RenSeq of these 20 selected restorer lines yielded between 0.27 to 2.24 Gb of circular consensus sequence (CCS) data per line with a mean of 1.40 Gb (Supplementary Table S6). De novo assembly using HiCanu produced 482 to 2122 contigs per line, with a mean of 1330. NLR annotation of the assemblies led to the identification of 288 to 1102 NLR contigs per line, with a mean of 646 (Supplementary Table S6), consisting of 151 to 573 partially (incomplete) annotated NLR contigs and 137 to 529 complete (full-length) NLR contigs per line.

Following SMRT RenSeq, we conducted an analysis across the 20 restorer lines to test the in vitro capture efficacy of the 600 K Triticeae NLR bait-library. In total, 35,199 baits aligned to a minimum of one line. Of these baits, 23,195 were shared with Lo7. On average, 15,796 ± 2910 (SD) baits aligned to the genomic sequence from each line, of which 60.9% were considered core, as they aligned to NLR sequences present in more than 12 lines; 31.1% were common baits that aligned to NLR sequences present in 5–12 lines, and 8.0% were rare baits that aligned to NLR sequences present in fewer than 5 lines (Supplementary Figure S3e). Across lines, 75.9% of the aligned baits were shared with Lo7. Baits were found to align with an average of 113 bp ± 14.9 (SD), with a mean alignment identity of 91.9% ± 4.3 (SD) (Supplementary Figure S3a,b). Approximately 0.5% of the baits aligned with less than 80% alignment identity. Each bait mapped to an average of 4 ± 7 SD unique NLR contigs, with a mean of 63 ± 82 (SD) unique baits per NLR contig (Supplementary Figure S3c,d; Supplementary Table S5).

### 3.6. K-Mer-Based Association Genetics RenSeq (AgRenSeq) Analysis

**Figure 4:**
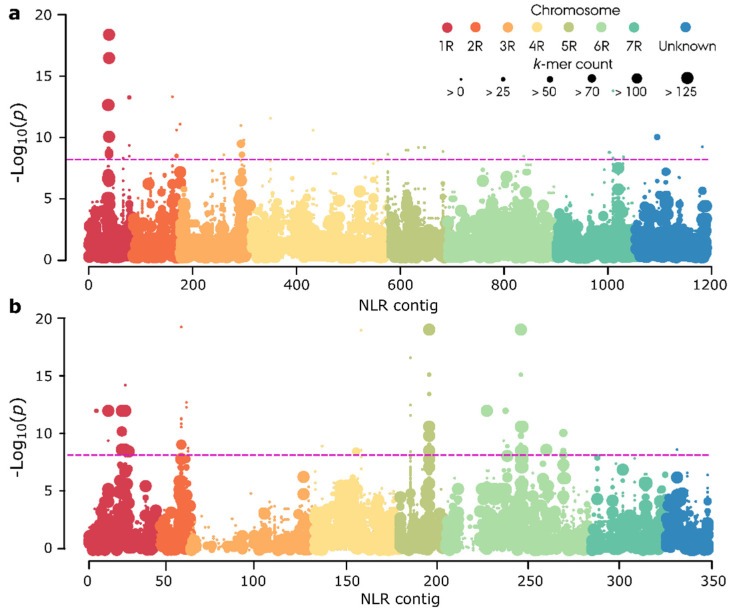
Identification of candidate leaf rust resistance NLR genes by AgRenSeq in 20 inbred rye lines. Each circle column on the x-axis represents an NLR contig from the SMRT RenSeq assembly of a resistant line anchored to the Lo7 rye reference genome and each dot on the y-axis represents a *k*-mer assigned a statistical association to resistance. (**a**) Resistant line RS13, phenotyped using isolate SPI-4. (**b**) Resistant line RS03, phenotyped using isolate SPI-6. The purple dashed line represents the Bonferroni-adjusted significance threshold based on number of *k*-mers.

To identify candidate NLR gene(s) for leaf rust resistance in the restorer panel, we performed association genetics on the *k*-mers (sub-reads) derived from the raw RenSeq data. To correct for false associations due to population structure, we applied a general linear model (GLM). The NLRs from a resistant line were anchored to the Lo7 reference genome to provide an ordered template for AgRenSeq (Figure 4). AgRenSeq identified three peaks, the most promising of which mapped to chromosome arm 1RS (−Log_10_(*p*) = 17.9) with a *k*-mer count of 932. The other two peaks pointed to chromosome arm 5RL (−Log_10_(*p*) = 18.7), with a *k*-mer count of 231, and to chromosome arm 6RL (−Log_10_(*p*) = 18.7), with a *k*-mer count of 160, as being potentially involved in resistance against leaf rust (Supplementary Figure S6). In total, we identified 25 NLR contigs harboring resistant-specific *k*-mers were identified across the shortlisted resistant lines (Figure 4a,b; Supplementary Table S8a).

### 3.7. Characterization of Candidate Genes Conferring Resistance to Leaf Rust Resistance

**Figure 5:**
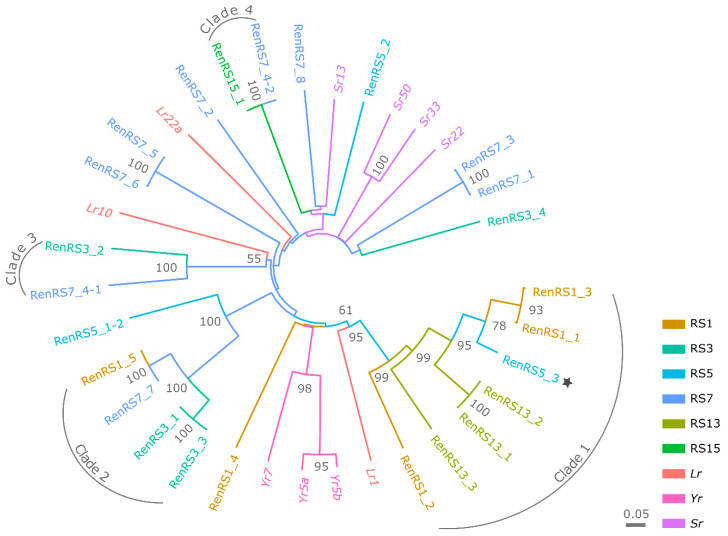
Phylogenetic relationship of candidate leaf rust resistance (*R*) NLR genes in 20 inbred rye lines. The tree was constructed using protein sequences, with bootstrapping values set as percentage recurrence of from 10,000 iterations shown for nodes with ≥50% recurrence. Candidate leaf rust *R* gene RenRS5_3 is marked by an asterisk. Examples of cloned wheat leaf rust (*Lr*), stripe rust (*Yr*) and stem rust (*Sr*) genes are included for comparison.

We sought to improve the quality of the NLR annotation by expanding contig size. To this end, we generated a manually curated reference-based local assembly for each of the 25 candidate leaf rust resistance NLR contigs obtained by AgRenSeq. Accordingly, we selected raw CCS reads mapping to each contig in the relevant resistant line harboring the candidate NLR contig and trimmed and assembled them. This manual assembly step increased the contig size by 2 kb ± 1.5 kb (SD), resulting in a mean contig size of 7.6 kb ± 1.8 kb (SD) (Supplementary Table S8). Out of the 25 contigs, 16 contigs were considered to be complete (full-length) NLR genes, with the remaining 9 being partial (incomplete). On average, the full-length NLRs encoded an NLR protein of 1147 amino acids (aa) ± 297 (SD). To distinguish unique NLR genes from homologous contigs within the panel, we performed a phylogenetic analysis based on the sequence alignment of the NLR proteins (Figure 5), leading to the identification of four clades comprising 15 NLR proteins and consisting of candidate Pr proteins shared between at least two resistant assemblies. Out of the 10 NLRs forming basal splits in the phylogeny, 5 belonged to the resistant line RS7.

**Figure 6:**
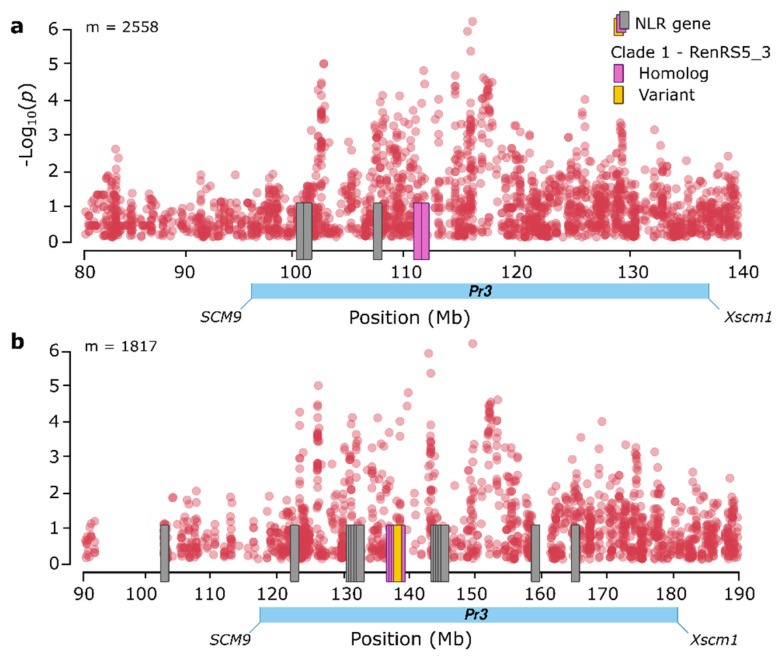
Visualization of SNP markers associated by GWAS with leaf rust resistance on chromosome arm 1RS in 92 inbred rye lines. (**a**) Lo7 reference genome with 2558 associated markers. (**b**) Weining reference genome with 1817 associated markers. The region harboring the known rye leaf rust resistance gene *Pr3* is marked as a blue bar based on flanking genetic markers *SCM9* and *Xscm1* [14]. The positions of annotated NLR genes are marked as vertical bars. NLR genes showing ≥80% sequence identity to the clade 1 member RenRS5_3 candidate *Pr* gene are in pink (‘Homolog’) and genes with ≥95% sequence identity are in orange (‘Variant’).

Clade 1 comprised seven full-length NLR contigs encoding proteins of 1414 to 1700 aa (Figure 5, Supplementary Table S8). All members were anchored to position 111.15 Mb on chromosome arm 1RS in the Lo7 reference genome, which also coincided with the genomic block showing the highest association with leaf rust resistance in the GWAS analysis, spanning from 101 to 117 Mb (Table 1, Figure 6a, Supplementary Table S4). This genomic region contained five NLR genes in the Lo7 reference genome. Of the 2558 SNP markers positioned within the region on Lo7 chromosome arm 1RS, 1817 markers mapped to the Weining reference over the 90–190 Mb interval. The region of the Weining reference genome had 19 NLR genes, of which 15 formed three larger clusters 5 NLR genes, each within the region spanning 130–145 Mb (Figure 6b). For comparative analysis with known leaf rust resistance genes mapping to the same chromosome arm 1RS, we mapped restriction fragment length polymorphism (RFLP) markers co-segregating with *Lr26* and *Pr3* to the Lo7 and Weining reference genomes. The *Xmwg68* marker co-segregating with *Lr26* mapped to 16.6 Mb in Lo7 and 19.5 Mb in Weining, with *Lr26* positioned distal to *Xmwg68* towards the telomeric tip, thus clearly excluded from the above candidate interval. The *SCM9* and *Xscm1* markers, co-segregating with *Pr3* and flanking the gene on either side, mapped to 96.7 and 137.6 Mb in Lo7 and to 118.1 and 184.1 Mb in Weining, respectively (Figure 6a,b).

Clade 2 consisted of four NLR contigs encoding near-identical canonical NLR proteins of 1383 to 1417 aa, distinguished by intraspecific polymorphisms in the distal end of the *C*-terminal LRR domain (Table 2, Supplementary Table S8). Clade 3 contained two NLR contigs encoding canonical NLR proteins of 950 and 1050 aa that exhibited considerable intraspecific polymorphisms. Clade 4 was defined by two identical NLR contigs encoding a noncanonical NLR protein of 713 aa with no N-terminal CC domain.

### 3.8. Characterization of Candidate NLR Genes Conferring Resistance to Leaf Rust on Chromosome Arm 1RS

**Figure 7:**
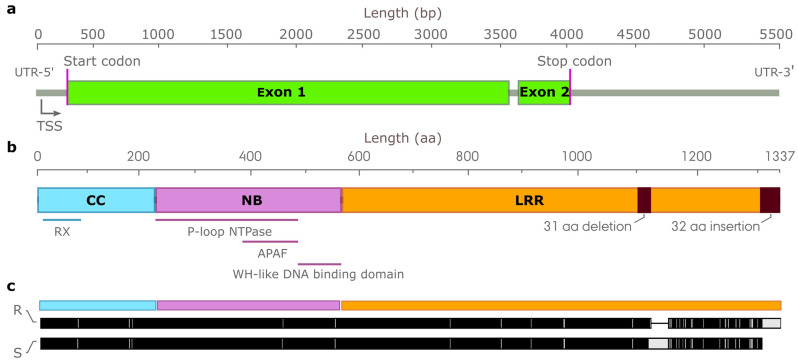
Characterization of the candidate leaf rust resistance nucleotide-binding leucine-rich repeat (NLR) gene RenRS5_3. (**a**) Gene structure, (**b**) NLR protein motif and domain structure, coiled-coil (CC), nucleotide-binding (NB), leucine-rich repeat (LRR)and (**c**) Alignment of RenRS3_5 resistant (R) and susceptible (S) variants with single amino acid substitutions marked as white lines.

We selected clade 1, with seven NLRs, as the most promising of candidate *Pr* genes for further characterization. The NLRs in this clade mapped to the region on chromosome arm 1RS, displaying the greatest association with leaf rust resistance (Figure 2), which also overlapped with the genomic location of the known leaf rust resistance gene *Pr3* (Figure 6a,b). To investigate gene presence/absence and sequence variation across the captured NLR complements from the 20 rye lines, we mapped all seven NLR contig members from clade 1 to the raw assemblies and scored and visualized the extent of sequence identity between putative homologs (Supplementary Figure S7). We then selected RenRS5_3 for further study as its complete annotation further facilitated its characterization. The RenRS5_3 contig was present (with ≥95% sequence identity) in 7 out of the 20 rye lines analyzed (Supplementary Table S9). Without transcript evidence, we predicted the most likely gene structure. We detected a putative transcription start site 45 bp into the contig, as well as two exons spanning the sequence from 199 to 3313 bp and 3404 to 4015 bp (Figure 7a). The corresponding NLR protein was predicted to have 1306 aa with an N-terminal CC domain from aa 17 to 225, a central NB-ARC domain from aa 226 to 586and a C-terminal LRR domain from aa 587 to 1306 containing 25 LRR motifs (Figure 7b). Manual reference-based assembly of RenRS5_3 in each of the seven lines carrying this NLR gene revealed two variants. One variant, denoted R, was conserved across the three resistant lines belonging to the IT_1_ group with 99.9% sequence identity; the second variant, denoted S, was conserved amongst susceptible or partially resistant lines belonging to the IT_2_ category with 97.8% sequence identity (Figure 3b). The two variants displayed a pairwise sequence similarity of 98.8% at the DNA level and were differentiated by 45 SNPs, 39 of which were situated in the genomic region encoding the C-terminal LRR domain. The variants encoded two NLR proteins showing 95.1% sequence identity, differentiated by 32 single amino-acid substitutions, a 31 aa deletion starting at aa 1105 and a 32 aa C-terminal extension of the LRR domain in the R variant (Figure 7c).

We identified NLR contigs with ≥97.8% similarity with the coding sequence of RenRS5_3 in three lines belonging to the IT categories IT_1_ and IT_3_. These contigs exhibited a more complex gene structure and encoded a noncanonical NLR protein of 1314 aa lacking the N-terminal CC domain and showing ≥87% sequence identity with the RenRS5_3 variants. Using the RenRS5_3 sequence as a query against the reference genomes, we detected two paralogous NLR genes in the Lo7 genome residing at 111.02 (Lo7_chr1R_nlr_64) and 111.15 Mb (Lo7_chr1R_nlr_65) and sharing 83.5–85.1% sequence similarity with RenRS5_3 (Figure 6a). In the Weining reference genome, RenRS5_3 aligned with a larger cluster of five paralogous NLR genes located from 137.1 to 138.9 Mb with ≥80.2% sequence similarity (Figure 6b). One of the NLR genes from Weining (Wei_chr1R_nlr_77) at 138.7 Mb showed 97.1% sequence identity with the R variant and 100% sequence identity with the ‘S’ variant.

## 4. Discussion

### 4.1. Leaf Rust Resistance Genes in Restorer Germplasm

In contrast to the predominant gene pools Petkus and Carsten deployed for hybrid breeding in rye, the restorer germplasm analyzed in this study boasts a high level of resistance to leaf rust (Supplementary Table S1) [2,15]. In relation to a recent study, we observed a strong correlation between resistance at the seedling stage and the adult stage [15], supporting the presence of major effect *R* genes in the restorer panel. Here, we discovered five intermediate-effect genomic regions on chromosome arms 1RS, 1RL, 2RLand 7RS, which are known to harbor *Pr* genes in rye [13,14,15]. Marker density was high, as the average intermarker distance was 25.5 kb in the assayed germplasm, which contributed to our ability to distinguish true associations characterized by a distinct marker peak from false-positive outliers [71]. While GWAS has become a routine strategy for the mining of *R* genes and genomic regions associated with resistance in crop species, establishing causality between an SNP marker and a given *R* gene of interest can be impeded by several factors, such as low genetic diversity and the rate of decay of linkage disequilibrium (LD) [32,33]. The restorer population used here was previously shown to exhibit a high genetic diversity, a relatively higher effective population size and lower linkage disequilibrium compared with the seed-mother population [72]. The rate of linkage decay in outcrossing species such as rye is often rapid and has been observed to occur within 4 kb in a similar hybrid breeding system in maize, providing potential opportunities for single-gene resolution at sufficient marker density [73]. While LD decay has not been investigated in the assayed restorer panel, the LD block surrounding the genomic region on chromosome arm 1RS appeared to span several Mb and contained multiple candidate *Pr* NLR genes (Figure 6). The small sample size and the potential for several rare or less prevalent *Pr* genes both reduce the phenotypic variance explained and, hence, the statistical power of GWAS [32,33,74]. Furthermore, several rye lines also displayed partial resistance against all six SPIs tested, suggesting the potential presence of broad-spectrum, slow-rusting *R* gene(s) or QTL. Slow rusting resistance is expressed by a susceptible infection-type of host reaction with reduced infection frequency and severity [75]. Although we failed to identify markers significantly associated with leaf rust resistance by GWAS, this approach nevertheless provided an important insight into the genetic architecture underlying resistance in the rye diversity panel phenotyped here and offered an opportunity for a comparative analysis with RenSeq.

### 4.2. Test of NLR Capture by the Bait Library

The 600 K Triticeae NLR bait-library was developed using NLR sequences from barley (*Hordeum vulgare* L.), hexaploid bread wheat (*Triticum aestivum* L.), tetraploid pasta wheat (*Triticum durum* L.), red wild einkorn (*T. urartu*), domesticated einkorn (*T. monococcum*) and three goat grass species (*Ae. tauschii*, *Ae. sharonensis* and *Ae. speltoides*) [42]. We first confirmed in silico that the bait library can perform a near complete capture of the NLR repertoire reported in the rye Lo7 reference genome [23]. During the course of our in vitro assessment, we observed a requirement for a minimum alignment identity between bait and target for efficient capture similar to that reported by Jupe et al. [37], thus supporting a key parameter used in the in silico analysis. In practice, only half of all baits aligned per line relative to our in silico analysis, suggesting a partial enrichment of the accessible NLR repertoire. This observation was further supported by the considerably lower number of NLR-annotated contigs per line (with a mean of 646 NLRs) compared to the 1167 NLRs present in the Lo7 reference genome. This discrepancy clearly falls outside the ≤40% expected range of accessional size variation of the NLR repertoire reported in previous RenSeq studies of *Ae. tauschii* and Arabidopsis [35,41]. Our assessment of bait library performance offered a glimpse into NLR capture across the Gülzow rye germplasm. However, additional data and analysis are required to explain the diverging success of NLR capture in this germplasm relative to the in silico analysis conducted on the Lo7 reference genome. Nonetheless, the immediate strength of RenSeq is the ability to mine for novel *R* genes in untapped genetic resources and wild-crop relatives without high-quality reference genomes [35]. This technique relies on the capacity of baits to align with a broad span of orthologous NLR genes with divergence sequences from the original NLR bait-library design. In the pioneering application of RenSeq by Jupe et al. [37], 68.5% of the enriched reads corresponded to genes that did not feature in the bait library design, thus demonstrating the plasticity of bait NLR capture.

### 4.3. K-Mer Association Genetics with SMRT RenSeq Data

The discovery of *R* genes using association genetics on subsequences depends on the detection of *k*-mer sequences specific to resistant lines, often differentiated by an SNV or InDel in the underlying NLR sequence. In contrast to the pioneering AgRenSeq study using Illumina short-read sequencing [35], here we used the updated PacBio^®^ SMRT sequel II HiFi system to achieve a higher precision in SNV and InDel calling [40]. When assessing the effect of sample size on *R* gene detection using AgRenSeq, Arora et al. [35] concluded that a panel of 80 diverse *Ae. tauschii* accessions was sufficient for detection of *SrTA1662 and Sr46*, two widespread stem rust *R* genes present in 42% of the accessions in the panel. However, increasing the sample size to 140 accessions allowed the detection of two additional rare variants present in as few as 5% of the tested individuals. In this study, we investigated whether the high accuracy of SMRT circular consensus sequences would enable the detection of *Pr* genes in a limited pool of 20 lines by AgRenSeq analysis. Using a multiple alignment-guided approach to group homologous NLR genes identified in multiple resistant lines, we successfully discovered four nonredundant candidate *Pr* genes. Comparative analysis of mapping positions was consistent with two genomic regions identified by GWAS on chromosome arms 1RS and 5RL.

### 4.4. Co-Discovery of a Candidate Pr Gene on Chromosome Arm 1RS

Serving as a proof-of-concept, one of the candidate *Pr* genes identified by AgRenSeq mapped to a pair of paralogous NLR genes in the Lo7 reference genome located at the center of a genomic region associated with leaf rust resistance on chromosome arm 1RS [14]. The underlying candidate *Pr* gene encoded a canonical NLR protein exhibiting sequence variation in the LRR domain, including a large deletion and a C-terminal extension of equal length. Loss-of-function and gain-of-function mutations are frequently discovered in the LRR domain, likely modulating the ability of the receptor to perceive the pathogen effector [76,77]. In flax (*Linum usitatissimum* L.), loss of a repeated unit within the LRR domain of *M* has been associated with the inactivation of rust resistance [78]. In the Weining reference genome, the paralogous NLR cluster has undergone considerable gene expansion, although one of the paralogs shares an identical sequence to that of the susceptible variant of the candidate *Pr* gene. NLR clusters generate new functional diversity and arise from tandem duplication events often followed by unequal crossover, intracluster rearrangements and gene conversion events [79]. In plants, several NLR clusters form the complex loci of multiple paralogous genes encoding NLR proteins with different resistance specificities against distinct races of a certain pathogen [80,81]. In maize (*Zea mays* L.), the rust resistance locus *Rp1* contains multiple copies of paralogous genes in various haplotype combinations carrying the *Rp1-A* and *Rp1-H* specificities [82]. The evolutionary capacity of complex NLR loci was highlighted in a study by Richter et al. [83], who identified four novel resistance specificities derived from recombination events within the maize *Rp1* complex. The candidate *Pr* gene discovered here may have arisen from such a cluster expansion in an ancestral line, followed by mutation events leading to a gain-of-function phenotype. This hypothesis may similarly explain the presence of the *Pr* gene paralog identified in the panel, accentuating the rapid generation of novel genetic variation in NLR clusters [79].

Chromosome arm 1RS from ‘Petkus’ rye has been widely deployed for the improvement of rust resistance in wheat through chromosomal translocation, carrying, amongst others, the two leaf rust *R* genes *Pr3* and *Lr26* [70,84]. We used the RFLP markers *SCM9* and *Xscm1*, which genetically co-segregate with *Pr3* [14], to physically delimit the genomic interval of the gene in the Lo7 and Weining reference genomes; the genomic coordinates of *Pr3* spanned the candidate *Pr* gene identified here by AgRenSeq. *Pr3* was initially discovered in a self-incompatible backcross family with the resistant parent of a Russian population of the rye variety ‘Jaroslavna’, segregating as a single dominant locus [14]. Whether the candidate *Pr* gene is *Pr3*, a paralog, a variant thereof, or merely resides in the same region as *Pr3* remains to be investigated.

The information we present here on rye NLR gene sequences provides a valuable genetic resource for the development of functional markers and for future genetic engineering [36]. With most breeding companies having invested in the infrastructure for MAS, molecular markers can now be easily integrated directly into commercial breeding programs [25].

In conclusion, we successfully demonstrated the use of SMRT AgRenSeq using high-fidelity long-read sequencing technology on a reduced sample size for the discovery of *Pr* genes in rye. In contrast to the predominant gene pools ‘Petkus’ and ‘Carsten’ used for hybrid breeding in rye, we observed a high level of leaf rust resistance in the tested germplasm. We identified five genomic regions situated on chromosome arms 1RS, 1RL, 2RL, 5RL and 7RS by GWAS with a high-density SNP array. While an in silico assessment of the 600 K Triticeae bait-library demonstrated a near-complete theoretical capture of the Lo7 NLR repertoire, our in vitro assessment showed partial target capture in the restorer germplasm. To explain this diverging NLR capture, additional data and analysis are required. We successfully identified four candidate *Pr* genes using SMRT AgRenSeq, including a canonical NLR gene whose genomic location overlapped with the top SNP associated with leaf rust resistance on chromosome arm 1RS. The nearly situated leaf rust resistance gene *Pr3* may be the underlying candidate *Pr3* gene or a paralog or variant thereof. In order to validate the candidate *Pr* genes identified in this study further investigations are required. Genetic validation could involve using a different type of genetically structured population, e.g., biparental, in combination with genetic markers to independently show a correlation between the segregation of markers and the resistant phenotype [85]. Functional validation could involve using knock out, EMS [86] or CRISPR-Cas9 [87] and/or transgenesis to express the functional unit in a susceptible background and confer the resistant phenotype to that background [88].

## Figures and Tables

**Table 1:** Genetic markers associated with leaf rust resistance identified from 92 hybrid rye restorer breeding lines by association genetics using 261,406 SNP array markers.

Chromosome	Position (Mb)	Marker ID	−Log_10_(*p*)	Phentypic Variance Explained (%)
1RS	115.55	AX-99251803	6.11	13.1
1RL	625.54	AX-99805135	4.50	9.3
2RL	818.90	AX-99478491	4.90	9.6
5RL	770.21	AX-99776626	4.49	9.3
7RS	26.93	AX-99684185	4.83	12.1

**Table 2:** Nucleotide-binding leucine-rich repeat (NLR) contigs associated with leaf rust resistance in four assigned clades based on phylogenetic relationships identified in resistant inbred rye lines. Contigs were mapped to the rye Lo7 reference genome.

Clade	NLR Contigs	Assemblies		Anchoring Position in Lo7	
		Resistant	SPI-Resistant	Chromosome Arm	Position (Mb)
1	7	RS1, RS3, RS5	RS13	1RS	111.15
2	4	RS1, RS3, RS7		5RL	792.53
3	2	RS3, RS7		NA	NA
4	2	RS7	RS15	5RL	807.97

SPI (single-pustule isolate); ‘NA’ (not anchored).

## Data Availability

The authors declare that the main data supporting the findings of this study are available within the article and its supporting information. All data and R scripts used to conduct the analysis and construct graphical outputs have been provided at https://doi.org/10.5281/zenodo.5725078 (accessed on 17 February 2022).

## Supplementary Materials

The following are available online at https://www.mdpi.com/article/10.3390/cells11081273/s1. Supplementary Table S1: Leaf rust severity of top-yielding hybrid rye (*Secale cereale* L.) and hybrid rye/population of rye mixtures tested in the Danish official trials in 2019 across nine locations. Supplementary Table S2: National sampling of *Puccinia recondita* f. sp. *secalis*, causative agent of leaf rust disease in rye (*Secale cereale* L.) in Denmark 2018–2019. Supplementary Table S3: Infection type response (0–9) of 92 inbred rye (*Secale cereale* L.) lines phenotyped using six geographically distinct Danish *Puccinia recondita* f. sp. *secalis* single-pustule isolates (SPI) collected in 2018 (SPI-1, SPI-2 and SPI-3) or 2019 (SPI-4, SPI-5 and SPI-6). Supplementary Table S4: Association of 600 K SNP markers with leaf rust resistance on chromosome arms 1RL, 2RL, 5RL and 7RL across 92 inbred rye (*Secale cereale* L.) lines phenotyped using *Puccinia recondita* f. sp. *secalis* single-pustule isolates. Supplementary Table S5: Metrics of in silico assessment of the 600 K Triticeae nucleotide-binding leucine-rich repeat (NLR) bait-library on the rye (*Secale cereal* L.) reference genome Lo7 and of in vitro assessment of 20 inbred rye lines analyzed using single-molecule real-time resistance gene enrichment sequencing. Supplementary Table S6: Metrics of single-molecule real-time (SMRT) resistance gene enrichment sequencing (RenSeq) analysis, assembly and NLR annotation of 20 inbred rye (*Secale cereale* L.) lines. Supplementary Table S7: Candidate leaf rust resistance nucleotide-binding leucine-rich repeat (NLR)-annotated contigs in resistant rye (*Secale cereale* L.) lines analyzed using resistance gene enrichment sequencing (RenSeq) identified using association genetics RenSeq (AgRenSeq) and AgRenSeq-GLM. Supplementary Table S8: Characteristics of nucleotide-binding leucine-rich repeat-annotated contigs associated with leaf rust resistance in 20 inbred rye (*Secale cereale* L.) lines analyzed using resistance gene enrichment sequencing and association genetics. Supplementary Table S9: BLASTN results between the candidate leaf rust resistance gene RenRS5_3 long-read resistance gene enrichment sequencing (RenSeq) raw circular consensus sequence data from 20 inbred rye (*Secale cereale* L.) lines. Data File S1. Genotype information for 261,406 informative 600 K SNP array markers in 92 inbred rye (*Secale cereale* L.) lines (.Rdata). Supplementary Figure S1: Geographical location of the origin of the six *Puccinia recondita* f. sp. *secalis* single-pustule isolates collected in Denmark in 2018 and 2019. Supplementary Figure S2: Manhattan plots for genome-wide association study (GWAS) for leaf rust resistance in 92 hybrid rye (*Secale cereale* L.) restorer breeding lines using 261,406 informative SNP markers mapped to the Lo7 reference genome. The entire germplasm was phenotyped for resistance against six distinct *Puccinia recondita* f. sp. *secalis* single pustule isolates (SPI) in a greenhouse with two replicate trials. The Bonferroni-adjusted significance threshold based on informative markers was 6.72. Supplementary Figure S3: Characteristics of NLR target sequencing in 20 inbred rye (*Secale cereale* L.) lines using a 60 K Triticeae NLR bait library. The reference genome Lo7 was included as an in silico reference with baits filtered for >80% alignment identity. (a) Distribution of bait alignment in lines with raw consensus sequence (CCS) data. (b) Distribution of bait alignment identity against raw CCS data. (c) Unique baits per NLR annotated contig. (d) Unique NLR annotated contigs per bait. (e) Total number of unique baits per breeding line and distribution of bait categories; ‘rare’ baits aligned to NLR sequence present in fewer than five lines, ‘common’ baits to 5–12 lines and ‘core’ baits to more than 12 lines. Supplementary Figure S4: Identification of resistance associated nucleotide-binding leucine-rich repeat (NLR) annotated contig across 20 inbred rye (*Secale cereale* L.) lines by association genetics resistance gene enrichment sequencing (AgRenSeq) analysis. (a) Standard AgRenSeq with candidate leaf rust resistance gene highlighted in red. (b), AgRenSeq with NLR contigs mapped to the Lo7 reference genome. (c), AgRenSeq-GLM. Supplementary Figure S5: Association genetics resistance gene enrichment sequencing (AgRenSeq) analysis for the identification of NLR contigs harboring resistant-specific *k*-mers in 20 inbred rye (*Secale cereale* L.) lines. Supplementary Figure S6: Association genetics resistance gene enrichment sequencing (AgRenSeq) GLM analysis for the identification of NLR contigs harboring resistant-specific *k*-mers in 20 inbred rye (*Secale cereale* L.) lines. Supplementary Figure S7: Distribution of alignment identity (%) of seven leaf rust resistance associated nucleotide-binding leucine-rich repeat (NLR) contigs belonging to clade 1 in 20 inbred rye (*Secale cereale* L.) lines long-read resistance gene enrichment sequencing (RenSeq) raw circular consensus sequence (CCS) data.
