# Supplementary material for: Discovery of Resistance Genes in Rye by Targeted Long-Read Sequencing and Association Genetics
Source: Cells. 2022 Apr 9;11(8):1273. doi: 10.3390/cells11081273 (PMC9032263; doi:10.3390/cells11081273)
Supplement: Supplementary file 1 [file cells-11-01273-s001.zip › cells-1658112-supplementary Figures.pdf]

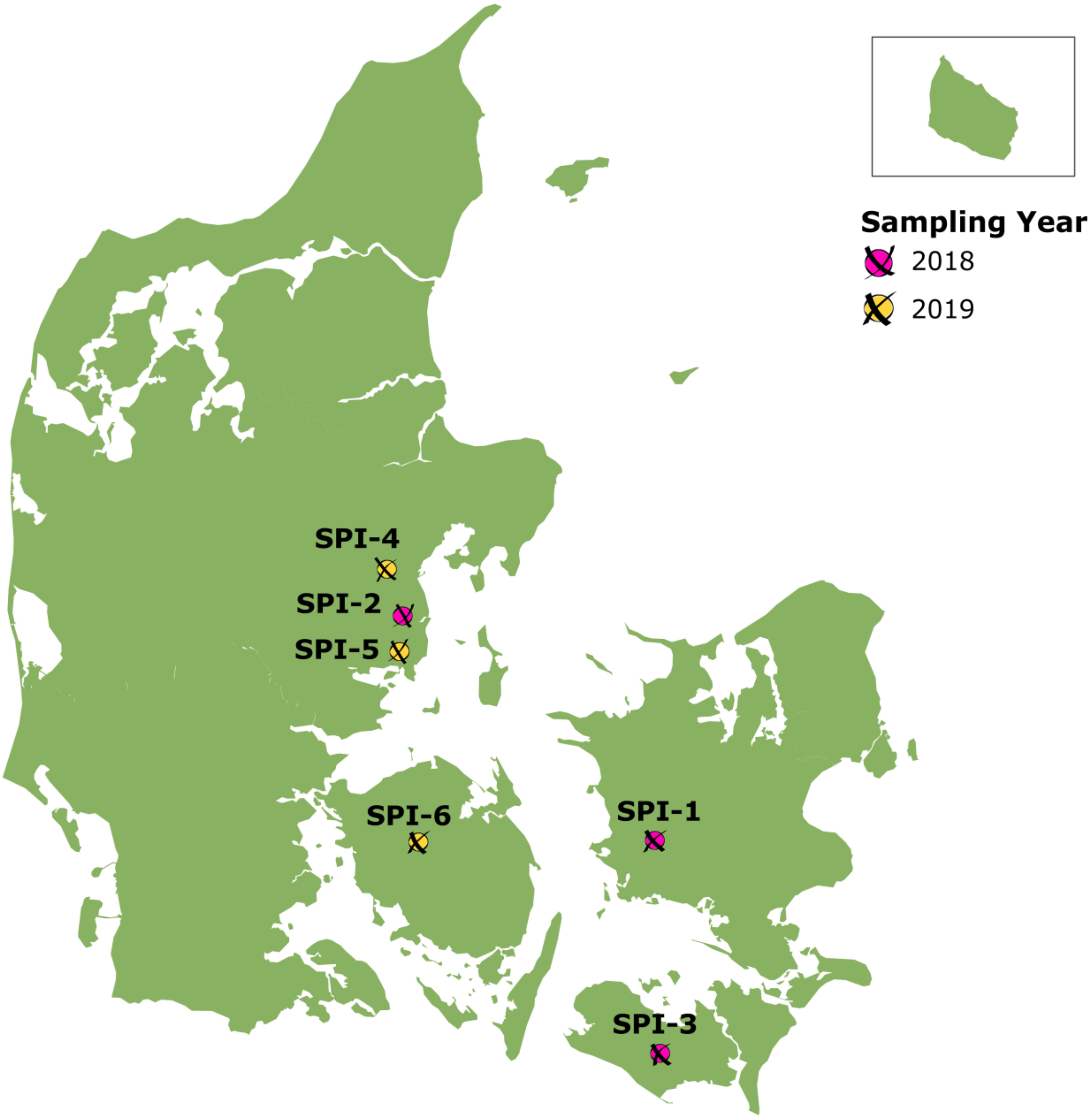

Figure S1. Geographical location of the origin of the six Puccinia recondita f. sp. secalis single -pustule isolates collected in Denmark in 2018 and 2019.

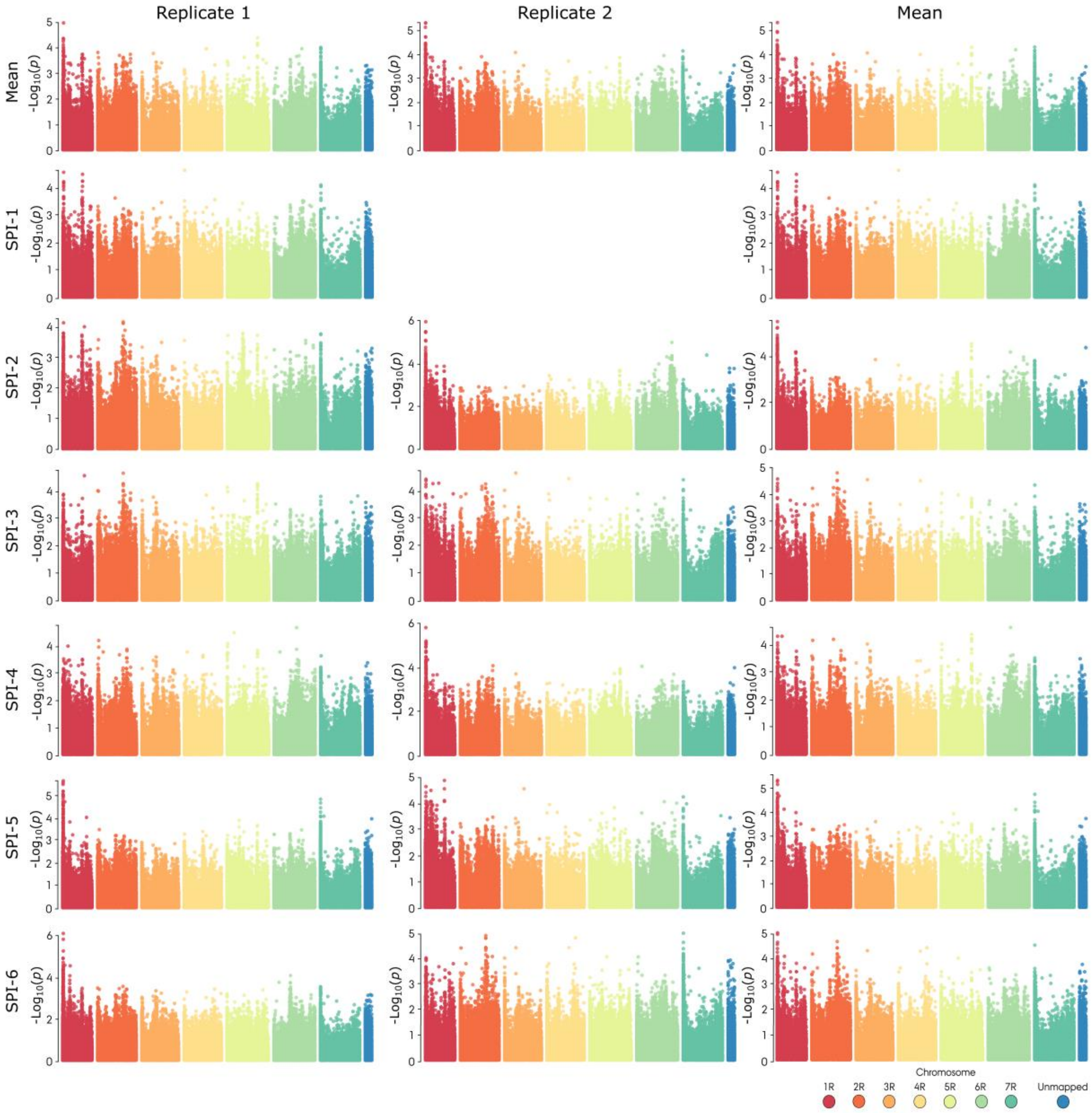

Figure S2. Manhattan plots for genome-wide association study (GWAS) for leaf rust resistance in 92 hybrid rye (Secale cereale L.) restorer breeding lines using 261,406 informative SNP markers mapped to the Lo7 reference genome. The entire germplasm was phenotyped for resistance against six distinct Puccinia recondita f. sp. secalis single pustule isolates (SPI) in a greenhouse with two replicate trials. The Bonferroni-adjusted significance threshold based on informative markers was 6.72.

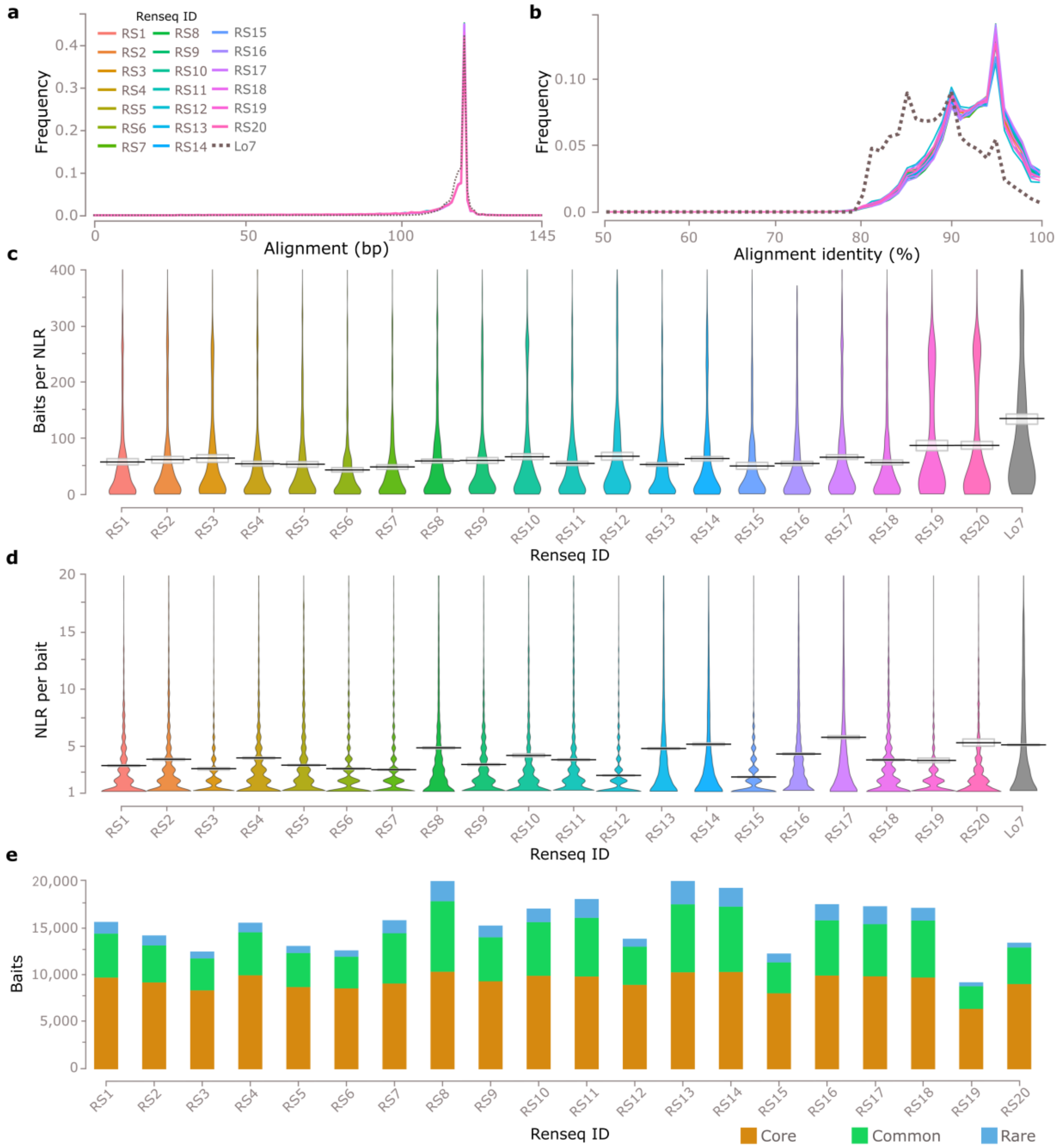

Figure S3. Characteristics of NLR target sequencing in 20 inbred rye (Secale cereale L.) lines using a 60K Triticeae NLR bait library. The reference genome Lo7 was included as an in silico reference with baits filtered for >80% alignment identity. (a) Distribution of bait alignment in lines with raw consensus sequence (CCS) data. (b) Distribution of bait alignment identity against raw CCS data. (c) Unique baits per NLR annotated contig. (d) Unique NLR annotated contigs per bait. (e) Total number of unique baits per breeding line and distribution of bait categories; 'rare' baits aligned to NLR sequence present in fewer than five lines, 'common' baits to 5–12 lines and 'core' baits to more than 12 lines.

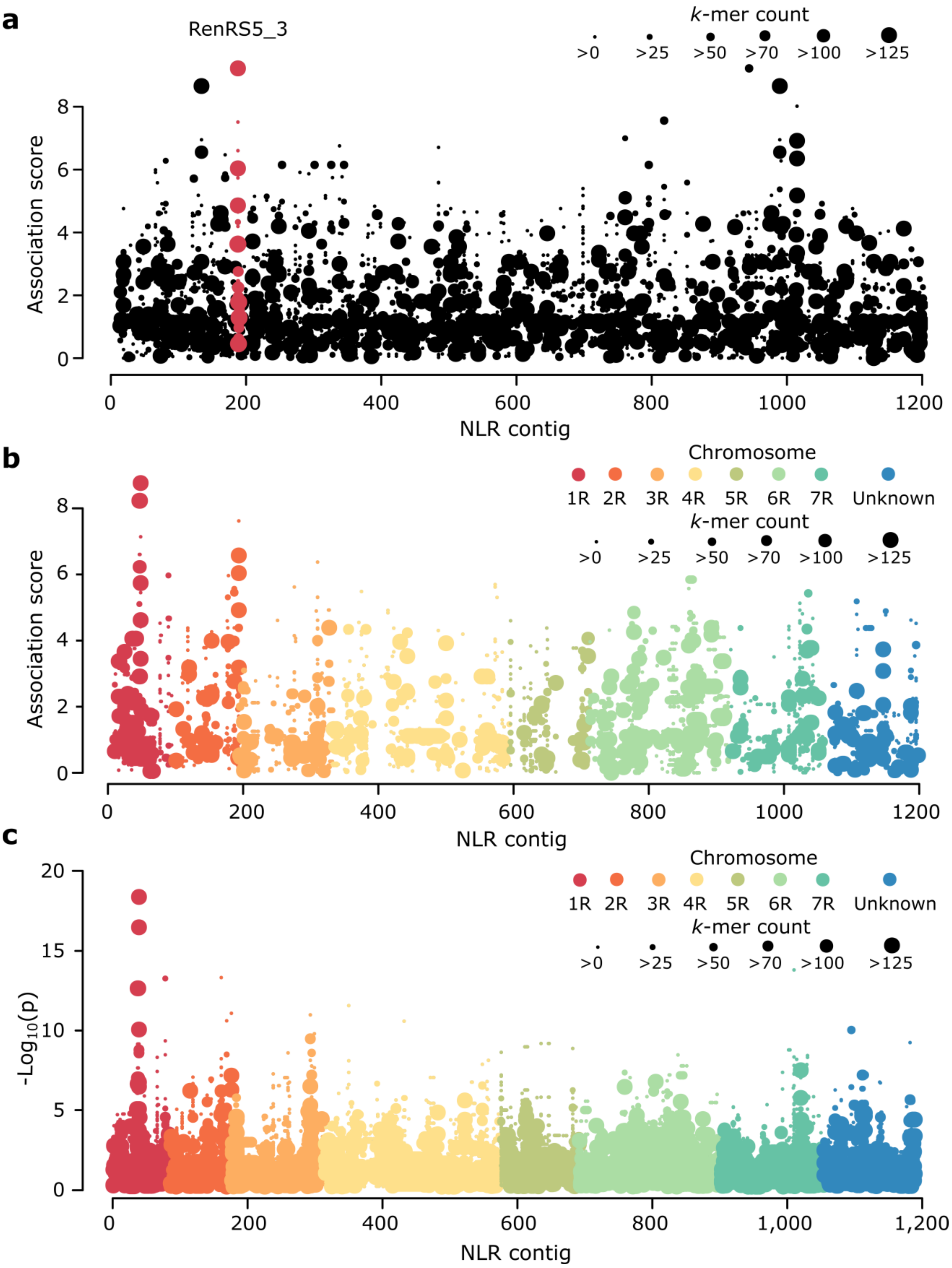

Figure S4. Identification of resistance associated nucleotide-binding leucine-rich repeat (NLR) annotated contig across 20 inbred rye (*Secale cereale* L.) lines by association genetics resistance gene enrichment sequencing (AgRenSeq) analysis. (a) Standard AgRenSeq with candidate leaf rust resistance gene highlighted in red. (b), AgRenSeq with NLR contigs mapped to the Lo7 reference genome. (c), AgRenSeq-GLM.

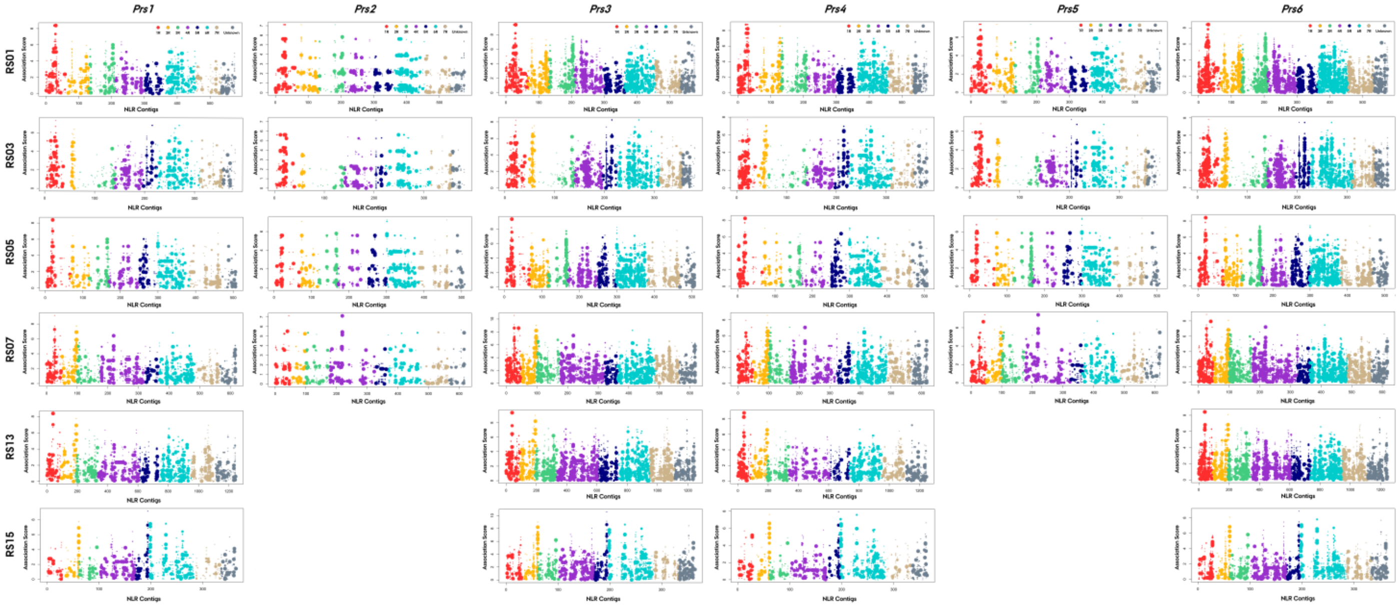

Figure S5. Association genetics resistance gene enrichment sequencing (AgRenSeq) analysis for the identification of NLR contigs harboring resistant-specific *k*-mers in 20 inbred rye (*Secale cereale* L.) lines.

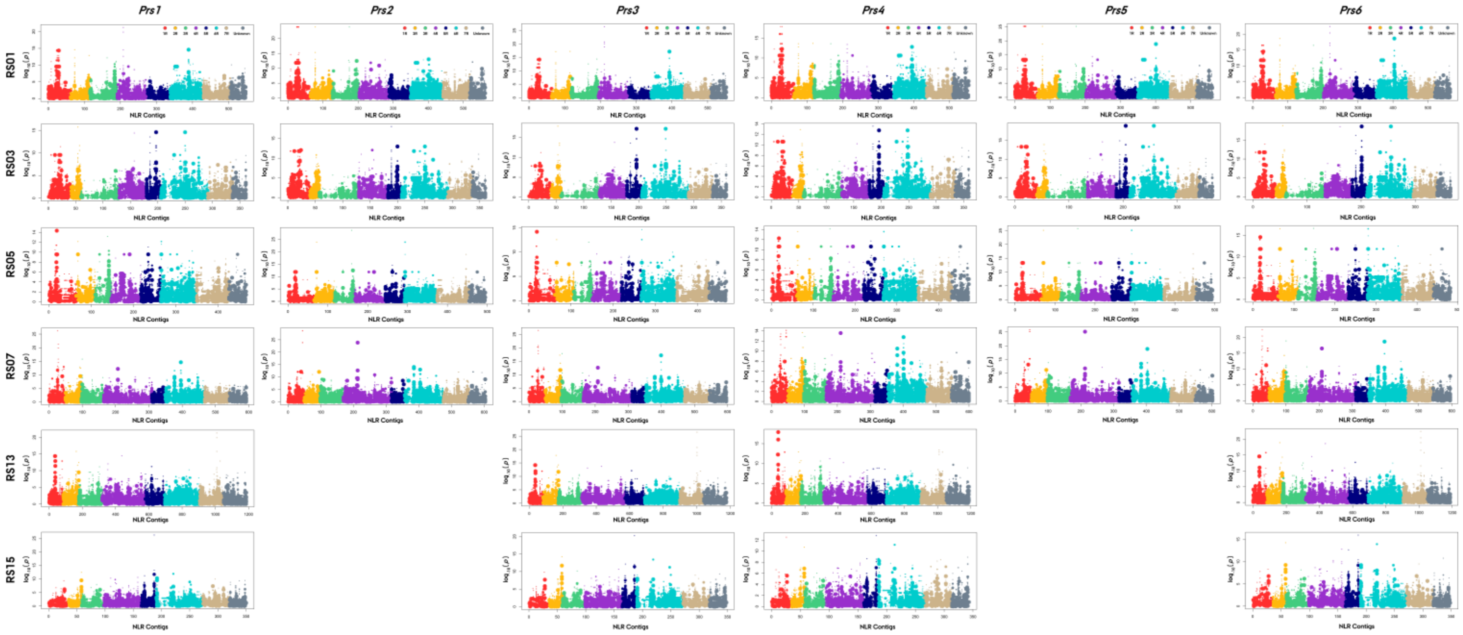

Figure S6. Association genetics resistance gene enrichment sequencing (AgRenSeq) GLM analysis for the identification of NLR contigs harboring resistant-specific *k*-mers in 20 inbred rye (*Secale cereale* L.) lines.

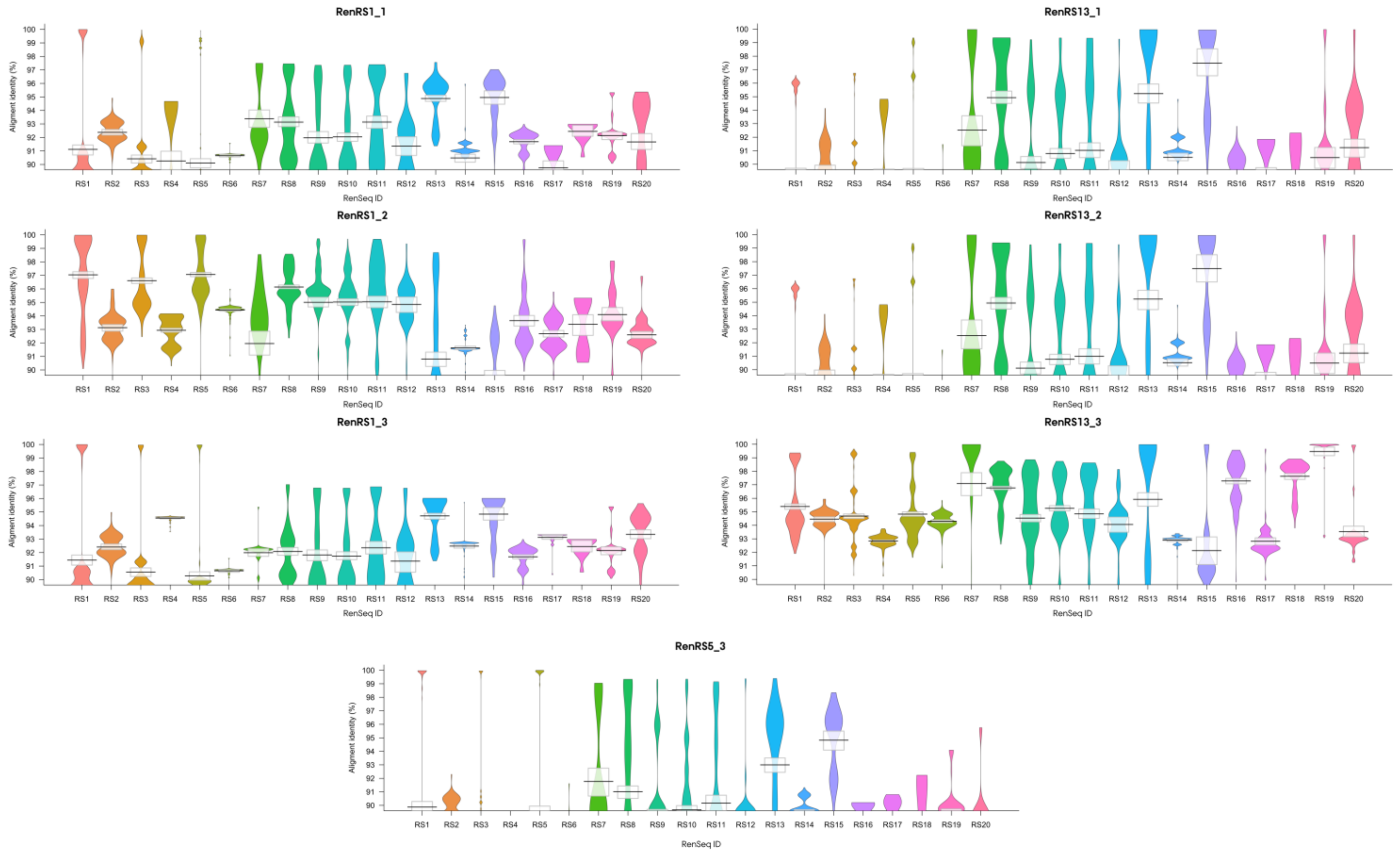

Figure S7. Distribution of alignment identity (%) of seven leaf rust resistance associated nucleotide-binding leucine-rich repeat (NLR) contigs belonging to clade 1 in 20 inbred rye (*Secale cereale* L.) lines long-read resistance gene enrichment sequencing (RenSeq) raw circular consensus sequence (CCS) data.
